# Utility of multimodality molecular profiling for pediatric patients with central nervous system tumors

**DOI:** 10.1093/noajnl/vdac031

**Published:** 2022-03-10

**Authors:** Prajwal Rajappa, Kenneth W Eng, Rohan Bareja, Evan D Bander, Melissa Yuan, Alisha Dua, Uday Bhanu Maachani, Matija Snuderl, Heng Pan, Tuo Zhang, Umberto Tosi, Iryna Ivasyk, Mark M Souweidane, Olivier Elemento, Andreas Sboner, Jeffrey P Greenfield, David J Pisapia

**Affiliations:** 1 Department of Neurological Surgery, Weill Cornell Medicine, New York, New York, USA; 2 Englander Institute of Precision Medicine, Weill Cornell Medicine, New York, New York, USA; 3 Department of Pathology, New York University Grossman School of Medicine, New York, New York, USA; 4 Department of Pathology and Laboratory Medicine, Weill Cornell Medicine, New York, New York, USA; 5 The Institute for Genomic Medicine, Nationwide Children’s Hospital, Columbus, Ohio, USA; 6 Department of Pediatrics, The Ohio State University Wexner Medical Center, Columbus, Ohio, USA

**Keywords:** brain tumor, next-generation sequencing, pediatric

## Abstract

**Background:**

As our molecular understanding of pediatric central nervous system (CNS) tumors evolves, so too do diagnostic criteria, prognostic biomarkers, and clinical management decision making algorithms. Here, we explore the clinical utility of wide-breadth assays, including whole-exome sequencing (WES), RNA sequencing (RNA-seq), and methylation array profiling as an addition to more conventional diagnostic tools for pediatric CNS tumors.

**Methods:**

This study comprises an observational, prospective cohort followed at a single academic medical center over 3 years. Paired tumor and normal control specimens from 53 enrolled pediatric patients with CNS tumors underwent WES. A subset of cases also underwent RNA-seq (n = 28) and/or methylation array analysis (n = 27).

**Results:**

RNA-seq identified the driver and/or targetable fusions in 7/28 cases, including potentially targetable *NTRK* fusions, and uncovered possible rationalized treatment options based on outlier gene expression in 23/28 cases. Methylation profiling added diagnostic confidence (8/27 cases) or diagnostic subclassification endorsed by the WHO (10/27 cases). WES detected clinically pertinent tier 1 or tier 2 variants in 36/53 patients. Of these, 16/17 SNVs/INDELs and 10/19 copy number alterations would have been detected by current in-house conventional tests including targeted sequencing panels.

**Conclusions:**

Over a heterogeneous set of pediatric tumors, RNA-seq and methylation profiling frequently yielded clinically relevant information orthogonal to conventional methods while WES demonstrated clinically relevant added value primarily via copy number assessment. Longitudinal cohorts comparing targeted molecular pathology workup vs broader genomic approaches including therapeutic selection based on RNA expression data will be necessary to further evaluate the clinical benefits of these modalities in practice.

**Key Points**

- RNA-seq was valuable in detecting targetable and/or diagnostically relevant fusion drivers and by informing therapeutic options based on outlier transcriptional readout.
- Methylation profiling provided critical WHO-sanctioned diagnostic refinement.
- WES provided added value primarily via copy number profiling, with most clinically interpretable SNVs/INDELs also covered by more targeted in-house NGS panels.

**Importance of the Study**

This study explores the clinical utility of WES, bulk RNA-seq, and methylation array profiling as an adjunct to more conventional diagnostic methods for pediatric CNS tumors in a representative single-institutional cohort, which can inform institutions on whether these modalities are worth employing. WES, RNA-seq, and/or methylation profiling identified clinically relevant calls in 94% of patients. 54/84 or 64% of these calls would not have been captured by our own standard modalities, including immunohistochemistry, FISH, or using our in-house targeted DNA/RNA NGS panel. RNA-seq detected outlier genes with high expression and rare fusion events, both offering the potential for rationalized treatment options. Methylation profiling was of utility, particularly in subclassifying medulloblastomas and ependymomas. While WES rarely detected clinically interpretable SNVs that would have been missed by our in-house targeted panels, it provided the benefit of broad copy number analysis and detected the variants of undetermined significance for future research.

More than 4600 children are diagnosed with primary central nervous system (CNS) tumors each year in the United States. The current standard of care for children with pediatric CNS disease has failed to significantly improve clinical outcomes for many of these patients, particularly those with high-grade, inoperable, recurrent, and/or metastatic CNS tumors.^1,2^ For example, in children with diffuse midline gliomas, more than 90% of patients die within 2 years after diagnosis, and the median survival rate is 9 months.^3^

Large-scale genomic studies of pediatric CNS tumors have revealed significant molecular heterogeneity within traditionally defined diagnostic entities, providing a rationale to reclassify pediatric tumors to offer more refined prognostic information and to design tailored treatment approaches.^4–9^ For example, the World Health Organization (WHO) now endorses molecular classification of medulloblastoma, the most common high-grade pediatric brain tumor, into at least 3 distinct molecular classes that, when supplemented with histological information, leads to improved prognostication^10–13^ and tailored therapeutics. Other examples include the discovery of recurrently altered oncogenic fusion drivers highly characteristic of specific histologically defined entities, such as the KIAA1549-BRAF tandem duplication seen in most pilocytic astrocytomas,^14^ and alterations that have already become a gold-standard diagnostic criterion in the WHO classification, such as “diffuse midline glioma, H3 K27-altered.” ^13,15^ Despite this evolving understanding of the molecular drivers of pediatric CNS tumors, most therapeutic regimens still rely on relatively nonspecific approaches that have existed for decades, such as targeting anti-neoplastic agents to rapidly dividing tumor cells.

Along with the explicit incorporation of molecular parameters into the WHO’s diagnostic rubric of CNS tumors, governmental and industrial entities, in addition to health care providers, increasingly recognize that the molecular stratification of a disease is a critical component of the diagnostic/prognostic/and therapeutic axis.^16,17^ For example, health insurance mechanisms are now considering next-generation sequencing (NGS) platforms for reimbursement.^18^

Studies demonstrating the incorporation of high-throughput molecular data into clinical care and its association with the detection of potentially actionable alterations that impact diagnosis, prognosis, and therapeutic options, are lacking. Here we explore the clinical utility of incorporating broad molecular testing as an adjunct to more targeted diagnostic approaches for pediatric patients with CNS tumors within a single institution and seek to define recommendations for the prioritization of diagnostic platforms. In this study, patients underwent whole-exome sequencing (WES) of both tumor and non-neoplastic DNA, with a subset of samples also undergoing bulk RNA sequencing (RNA-seq) and/or methylation array profiling. In this prospective, observational case series, we identify cancer-relevant somatic alterations, driver mutations, gene fusions, relapse-specific mutations, and epigenetic signatures over multiple platforms and demonstrate a proof of principle for a multimodality approach to pediatric tumor assessment. At the same time, we uncover important limitations and caveats to some of the assays employed. Our data suggest that by carefully selecting a core set of diagnostic modalities, physicians will be better equipped to offer patient-specific clinical decisions with the potential to demonstrate improved outcomes in the future.

## Methods

### Patient Population and Characteristics

Informed consent (and assent where applicable) was obtained according to an established Research for Precision Medicine protocol approved by the Institutional Review Board of Weill Cornell Medicine (IRB protocol #1305013903) from 53 pediatric patients (0-19 years at the time of diagnosis) with primary tumors of the CNS and their families.

### Precision Medicine Approach for Materials Collection

Intraoperatively, institutional biorepository personnel immediately transported freshly resected tissue to surgical pathology. Following intraoperative assessment by a board-certified neuropathologist, tissue was allocated into frozen aliquots for WES and RNA-seq. Tissues were analyzed for Germline DNA using peripheral blood or buccal swabs as previously reported.^19–22^ For patients with recurrent disease who were initially treated at an outside hospital, archival formalin-fixed paraffin-embedded (FFPE) tissue was used when available for comparative genomic analyses.

### Whole-Exome Sequencing and Germline Analyses

WES was performed on matched tumor and non-neoplastic tissues using previously described techniques.^19,20,22,23^ DNA was extracted from macrodissected FFPE or fresh frozen tumor tissue along with peripheral blood lymphocytes or buccal tissue using the Promega Maxwell 16 MDx. The Agilent HaloPlex kit, an enzymatic capture method, was used for library preparation. Both neuropathology review and CLONET software (OMICS_07304), a computational tool to estimate ploidy and tumor content, estimated tumor content. An Illumina HiSeq 2500 system (2 × 100 bp) was used to sequence all samples. A total of 21 522 genes were analyzed with an average coverage of 90× and 94× for tumor and non-neoplastic specimens, respectively. All bioinformatics analyses were performed according to the Clinical Laboratory Improvement Amendments (CLIA)-approved Whole-Exome Sequencing Test For Cancer-EXaCT-1-pipeline version 09.^20^

Germline variants were screened based on our most recent germline pipeline.^19,20^ For the purpose of the study, we included only variants in 152 cancer-associated germline genes. All candidate pathogenic germline variants detected were manually reviewed with the Integrative Genome Viewer.^24^

### RNA Sequencing and Analyses

RNA-seq and data processing were performed as previously described^22^ on samples from 28 patients. In short, RNA was extracted from frozen material for RNA-seq using the Promega Maxwell 16 MDx instrument (Maxwell 16 LEV simplyRNA Tissue Kit, cat. # AS1280). Specimens were prepared for RNA-seq using TruSeq RNA Library Preparation Kit v2 as previously described.^20^ The Agilent Bioanalyzer 2100 (Agilent Technologies) verified RNA integrity. cDNA was synthesized from total RNA using Superscript III (Invitrogen, Thermo Fisher Scientific Corporation). Sequencing was then performed on the HiSeq 2500 (Illumina) as paired-ends.^22^ Similar to the original analysis, STAR_2.4.0f1 independently mapped all reads against the reference genome for sequence alignment against the human genome sequence (UCSC build hg19). Cufflinks (2.0.2)^25^ was used to estimate the expression values (FPKM) and GENCODE v19 GTF file for annotation. RStudio (1.0.136) with R (v3.3.2) and ggplot2 (2.2.1) performed statistical analyses and generated figures. The gene counts from the htseq-count script and the DESeq2 Bioconductor package identified differentially expressed genes. We used STAR-Fusion (STAR-Fusion_v0.5.1) for detecting gene fusions.^26^ We cut off Junction Reads (>2) and Spanning Pairs (>1) to filter out false-positives. We visualized the fusions by creating a network of fusions using the OmicCircos package (Bioconductor). CIBERSORT and xCell deconvoluted transcriptomes.^27,28^ Gene expression outliers representing potential therapeutic targets were assessed by calculating *Z*-scores for a list of 74 cancer-related genes generated from the intersection of the Sanger database and DrugBank (https://www.sanger.ac.uk/science/tools/gdsc-genomics-drug-sensitivity-cancer and https://www.drugbank.ca/).

### Methylation Array (450 K) and Analyses

Genome-wide methylation profiling of 27 pediatric CNS tumors was performed using the Illumina Infinium HumanMethylation450k BeadChip as previously described.^11^ Bioinformatic analyses were conducted using the ChAMP package (version 2.12.0) from the R Bioconductor software. A numerical beta-value for each probe was used as the methylation level in the principal component analyses (PCA). R was used to perform functional and PCA analyses in all probes. The top 1% most variable probes (MVPs), top 5% MVPs, and top 10% MVPs were reported and clustered (probes were ranked by the standard deviation across all 27 samples).

### Variant Calls Annotation and Data Reporting

Our WES computational pipeline detects single-nucleotide variants (SNVs), small insertions and deletions (INDELs), and somatic copy number alterations (CNAs). Those alterations are classified into different tiers according to our Precision Medicine Knowledge Base (PMKB).^29^ The molecular pathology teams reviewed each case to evaluate the “borderline” variant calls, that is, those near the limit of detection of the assay. Once this process was completed, a report was then drafted where variants were stratified into 3 tiers with respect to an estimated clinical yield. Tier 1 variants were considered either targetable with well-established targeted therapies (eg, drugs targeting *BRAF* V600E or *NTRK* alterations) and/or diagnostic according to WHO criteria. Tier 2 alterations were considered either highly characteristic of a particular tumor entity (though not considered a gold-standard diagnostic criterion per se by WHO guidelines), represented known cancer-related alterations with likely functional significance, variants with potential prognostic significance independent of the diagnosis, or were considered potentially targetable alterations based on gene expression of genes associated with targetable pathways. Tier 3 alterations comprised variants of unknown significance that did not meet the criteria for tiers 1 or 2. Selected cases, such as those that demonstrated tier 1 alterations, were presented at the Englander Institute for Precision Medicine Tumor Board at Weill Cornell Medicine.

## Results

### Precision Medicine Approach and Clinical Integration

**Figure 1.**
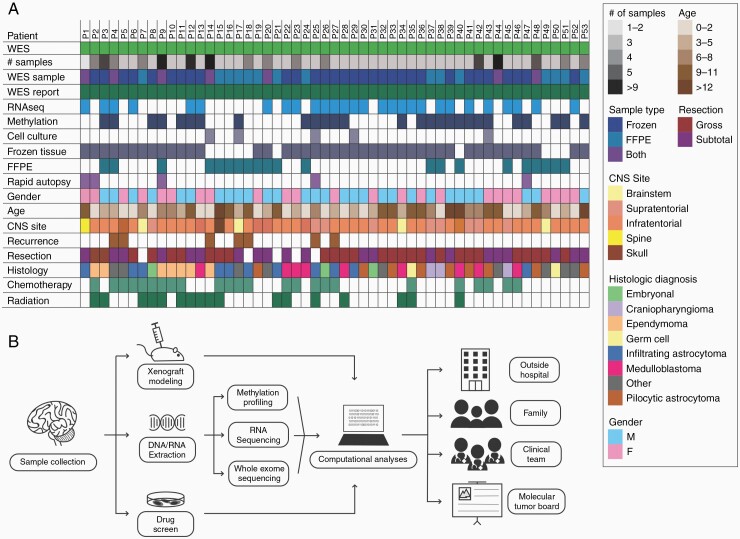
Cohort summary and program flow diagram. (A) Overview of patients enrolled in pediatric precision medicine pathway with respect to patient demographics, associated clinical parameters, diagnostic class, number of aliquots/samples and tissue types available for precision medicine analysis, tissue types used for analysis (WES sample), and classes of testing completed. (B) Flow chart demonstrating algorithm for precision medicine from initial sample collection from the operating room to sequencing platforms offered including option to perform drug screens and xenograft modeling for select patients. Genomic variants are ultimately validated at the EIPM computational core and reports are disseminated to outside providers, families, and the clinical team. Patients with clinically relevant variants are also presented to a multidisciplinary tumor board. WES, whole-exome sequencing; FFPE, formalin-fixed paraffin-embedded; EIPM, Englander Institute for Precision Medicine. *Unless otherwise specified, filled boxes represent “yes” and empty boxes represent “no.”.

In this study, we report data from a multi-parametric set of molecular analyses over our initial set of 53 pediatric patients with CNS tumors. Slightly more patients were male (54.7%). 86.8% of patients enrolled at the time of the initial diagnosis. A diversity of tumor entities was represented, including CNS embryonal tumors, craniopharyngiomas, ependymomas, germ cell tumors, infiltrating astrocytomas, medulloblastomas, and pilocytic astrocytomas (Figure 1A). An overview of our cohort characteristics, including demographics, cancer diagnostic class, number of samples per subject, assays performed, and treatment provided (chemotherapy or radiation) are also summarized (Figure 1A). After sequencing, neuropathologists and molecular pathologists performed and reviewed computational analyses, variant annotation, and tumor classification. Finally, the treating physicians and family received precision medicine reports summarizing the available data. The precision medicine workflow is illustrated in Figure 1B.

### Somatic DNA Alterations

**Figure 2.**
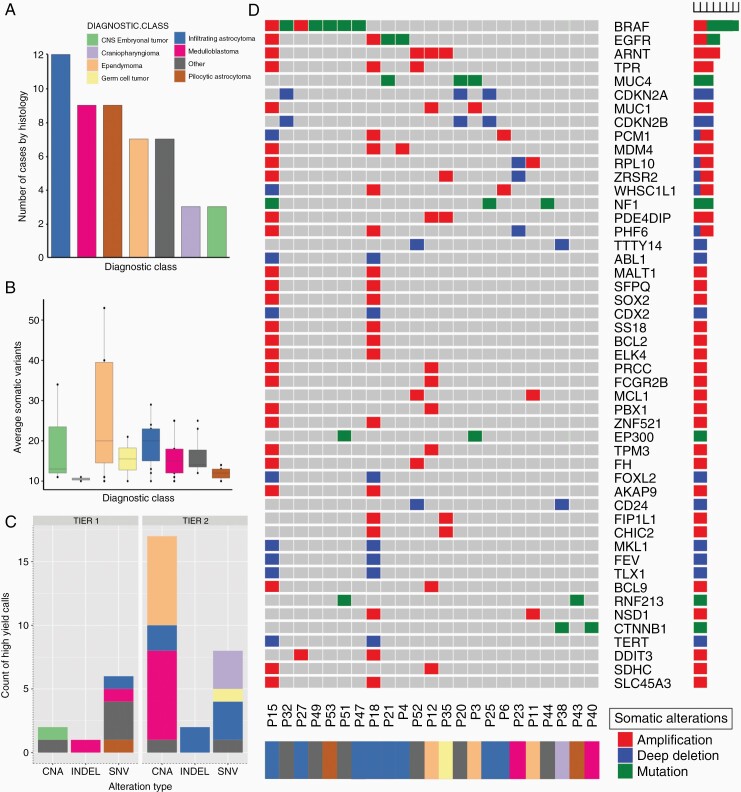
Summary of critical calls. (A) Number of cases by specific diagnostic class in our cohort (n = 53). (B) Number of somatic variants by diagnostic class detected with WES. Infiltrating astrocytomas harbored the highest mean alterations while ependymomas demonstrated the most variable range. (C) Summary of tier 1 and tier 2 calls by alteration type (CNA, copy number alteration; INDEL, insertion/deletion; and SNV, single-nucleotide variations). (D) Global overview of our cohort and recurrent alterations detected from WES.

We performed WES on 53 matched tumor and non-neoplastic specimens (peripheral blood or buccal swab) from distinct anatomic locations and diverse tumor types (rows 13 and 16 of Figure 1A). The mean depth of coverage was 90× and 94× (non-neoplastic and tumor tissue, respectively). In this cohort, infiltrating astrocytomas comprised the largest group by diagnostic class (n = 12) followed by medulloblastoma (n = 9) and pilocytic astrocytoma (n = 9) (Figure 2A). CLONET software^30^ determined tumor purity from WES data, which ranged from 17.68% to 99.70%. From WES analyses, the highest tumor mutational burden (TMB) was observed in infiltrating astrocytomas while craniopharyngiomas had the lowest TMB. Similarly, based on WES data, infiltrating astrocytomas (Figure 2B, blue) had the highest mean number of somatic variants followed by ependymomas (Figure 2B, peach) but also had a wide range. Craniopharyngiomas (Figure 2B, lavender) had the fewest mean somatic variants.

Across the 53 cases, 9 WES calls were considered tier 1 calls (7 SNVs/INDELs and 2 CNAs), and 27 were considered tier 2 (10 SNVs/INDELs and 17 CNAs) (Figure 2C; also see Supplementary Table 1). Despite the diversity of tumor types present in this cohort, we observed recurrent somatic alterations from WES (Figure 2D). *BRAF* alterations were the most frequent overall and *BRAF* V600E mutation, in particular, a tier 1 alteration with important therapeutic implications was detected in 5 cases. Importantly, *BRAF* V600E was detected in 3 cases with otherwise nonspecific histopathological diagnoses including “Infiltrating astrocytoma,” “Astrocytoma, NOS,” and “Low grade astrocytoma, NOS,” thus providing not only therapeutic information but crucial diagnostic information in revealing a putative oncogenic driver, thereby making it much less likely that other diagnostically relevant drivers were being missed (eg, an H3 alteration in the case of the infiltrating astrocytoma). The other 2 cases were that of a histologically typical pilocytic astrocytoma and pleomorphic xanthoastrocytoma (PXA) (Supplementary Table 1). Additional recurrently altered genes included *EGFR* and *ARNT* alterations (n = 4 each). In terms of copy number assessment, WES provided prognostically relevant copy number information independent of the diagnosis (tier 2) in 14/53 cases, including 1q assessment in ependymomas and chromosome 17 assessment in medulloblastoma (Supplementary Table 1). In additional cases, copy number assessment afforded by WES revealed critical diagnostic information (tier 1); for example, in an otherwise poorly described CNS embryonal tumor, focal amplification of 19q13.42 was detected, which is entity-defining for “embryonal tumor with multilayered rosettes, C19MC-altered.” Additional critical CNAs included detection of *SMARCB1* loss in a case of chordoma, leading to a diagnosis of poorly differentiated chordoma in a case with poor characterization of INI1 expression by immunohistochemistry (IHC), and *CDKN2A/B* loss in the case of PXA mentioned above.

### Germline Findings

We potentially identified pathogenic germline variants in 7 of the 53 patients (13.2%). These germline frameshift variants included *PTCH1*, *COL7A1*, *RHBDF2*, *UROD*, *NHP2*, *ERRC5*, and *XPA*. Germline inhibition of the *PTCH1* tumor suppressor gene, reported in a subset of medulloblastomas, suggests an essential pathway for tumor development and progression.^10^ Moreover, this *PTCH1* variant would not have been covered by our current in-house 500-gene NGS panel.

### Transcriptomic Analysis

**Figure 3.**
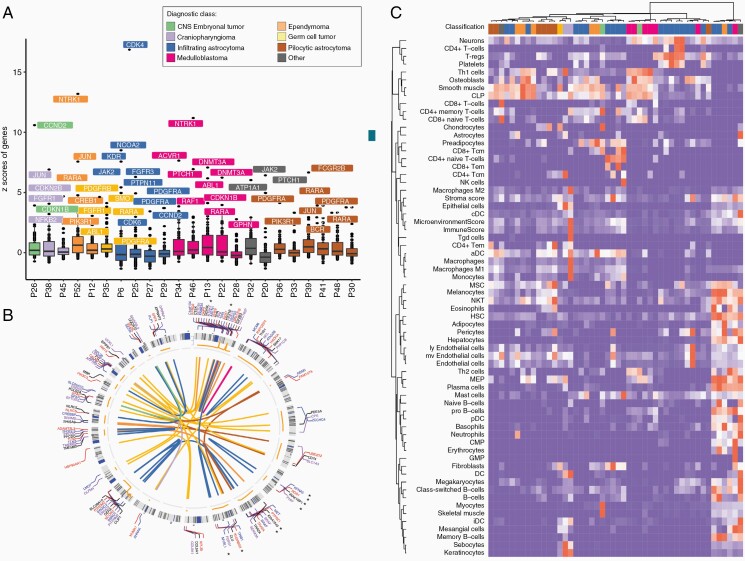
Utility of RNA interrogation. (A) Outlier gene expression profile by diagnostic class demonstrates overexpressed targetable genes. (B) Network fusion plot demonstrates detected fusions including clinically relevant variants such as MYBL1-KHDRBS3, KIAA1549-BRAF, and KIF21B-NTRK1 marked by asterisk (*). (C) Computational deconvolution from bulk RNA sequencing of select cases from our cohort (n = 28) demonstrating associated TME (tumor microenvironment) cell signatures specific to diagnostic class based on unsupervised clustering of gene expression profiling.

We performed RNA-seq on frozen tissue samples from 28 patients in our cohort. RNA-seq demonstrated outlier expression of potentially druggable gene targets (see Methods section) in 68% of sequenced cases (19/28). In this analysis, known drug target genes with the highest expression were selected relative to the entire cohort of samples in the Englander Institute of Precision Medicine, as described in the section Methods. Potentially targetable outlier genes included *CDK6*, *CDK2NB*, *FGFR1*, *JAK2*, *NFKB2*, *NTRK1*, *PDGFRA*, and *PIK3R1* (Figure 3A). RNA-seq also revealed actionable gene fusions in 2 additional cases (*NTRK1-TRP* and *KIF21B-NTRK1*). Of note, the case found to harbor the KIF21B-NTRK1 fusion also demonstrated outlier expression of NTRK1, providing multiple data points supporting the targeting of the NTRK in that case. Additional fusions from this cohort including clinically relevant fusions observed upon RNA-seq included *IRAK1BP1-KPNA5*, *PHIP-ROS1*, *MYBL1-KHDRBS3*, and *KIAA1549-BRAF* (Figure 3B). In particular, the *MYBL1* fusion transcript detected was the only putative oncogenic driver in this case, over all platforms including WES, and was subsequently reported as a recurrent driver in a subset of pediatric infiltrating gliomas.^31^ Moreover, the alteration is now entity-defining according to new WHO criteria.^13^ In contrast, a fusion such as the *IRAK1BP1-KPNA5* has not been detected by our group in any additional cases and has not been reported previously, thus its functional significance remains unknown and further experiments would be required to characterize its oncogenic potential. To further interrogate the potential utility of bulk RNA-seq across tumors, we performed computational bulk tumor deconvolution analysis to assess the tumor microenvironment.^32–39^ Gene expression profiles related to immune cell signatures broadly clustered differentially, with lower-grade gliomas and ependymomas in a similar group and the majority of medulloblastomas and high-grade infiltrating astrocytomas appearing to cluster in a distinct group (Figure 3C). This type of analysis provides a proof of principle that bulk RNA-seq, even over relatively small but diverse cohorts, has the potential to provide biological insights into the interaction between tumor and non-neoplastic elements in pediatric tumors of the CNS.

### Epigenomic Analysis

**Figure 4.**
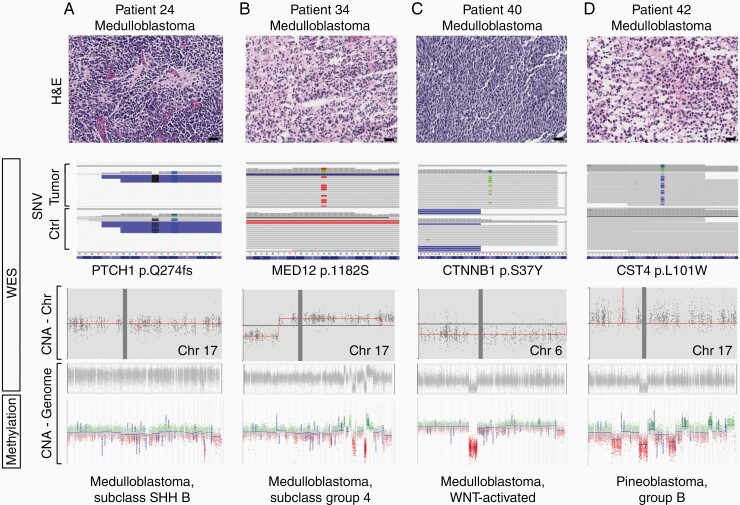
Integrated profiling of medulloblastoma. Methylation array (450K) offered to select patients (n = 25) enhanced precision diagnostic and molecular characterization. In patients 24, 34, and 40, variants detected upon array helped categorize these distinct medulloblastomas into their molecular subgroups (SHH, subgroup 4, WNT-activated). Patient 42 was histologically diagnosed as a medulloblastoma, however, based on the methylation classifier data, this tumor was reclassified as a pineoblastoma, group B with a high calibrated score (0.99). Scale bar = 50 µm.

Tumor tissue (based on availability) from 27 patients was assayed for DNA methylation (Figure 1A) using the 450K bead array, a diagnostic tool with proven utility.^11^ By using PCA based on methylation profiles alone, we demonstrated that even a relatively small cohort showed disparate entities clustering robustly in accordance with their histological features (Supplementary Figure 1). Furthermore, we demonstrated the utility of methylation classification in distinguishing between specific molecular subclasses of medulloblastoma (as seen in patients P24, P34, and P40) (Figure 4). Specifically, each histologic diagnosis of medulloblastoma was then molecularly subclassified into SHH B, group 4, and WNT-activated tumors for patients P24, P34, and P40, respectively (Figure 4), which conferred risk stratification and ultimately may guide neuro-oncology decision making for pursuing standard or aggressive treatment approaches. Patient P28 was also subclassified as a group 4 medulloblastoma (Supplementary Table 1). In 1 case, a tumor was reclassified from medulloblastoma to pineoblastoma based on the methylation profile, even after 3 independent reviewing institutions had rendered a diagnosis of medulloblastoma (patient P42; Figure 4). Methylation profiling offered additional class information or refined diagnostic precision in 5 additional non-medulloblastoma cases (9% of cases in this cohort) including a “CNS embryonal tumor, NOS” that classified most closely to a FOXR2-altered tumor (calibrated score of 0.717; patient P26); and a patient whose tumor demonstrated histological features of an infiltrating astrocytoma and was shown to have a methylation profile most closely resembling that of an adult-type IDH-wildtype glioblastoma (calibrated score of 0.88; patient P4).

### Clinical Utility of Multimodality Diagnostics Over Conventional Approaches

**Figure 5.**
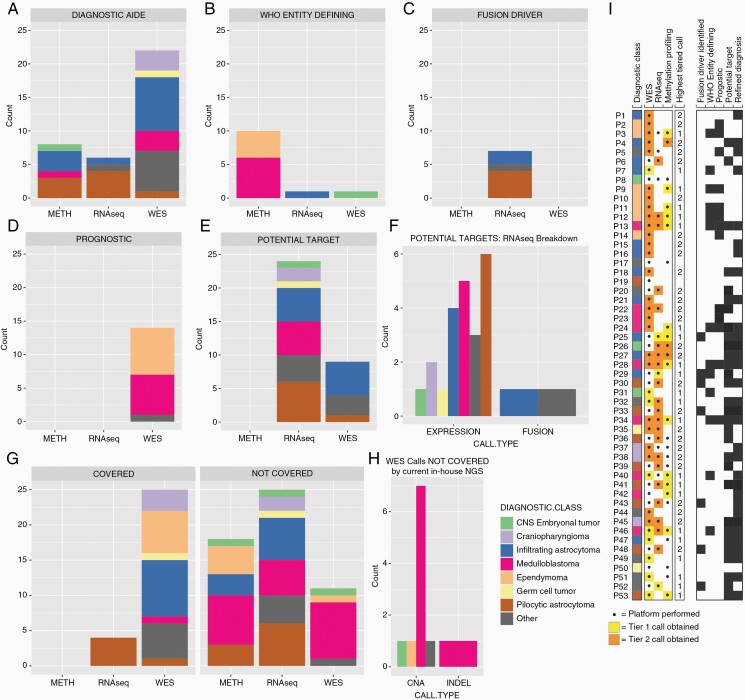
Platform-based clinical utility. (A–E) Count of calls from methylation analysis, RNA-seq, or WES that (A) aided in diagnosis; (B) classified as WHO entity-defining; (C) identified fusion drivers; (D) aided in prognostication; or (E) were potentially targetable. (F) Counts of potential targets identified by RNA-seq calls that were identified based on expression level vs fusion. (G) Count of methylation/RNA-seq/WES calls that would have been covered (left) or not covered (right) by current in-house NGS testing. (H) WES calls not covered by current in-house NGS testing, divided by call type (CNA, copy number alteration vs INDEL, insertion/deletion). (I) Clinical utility heatmap demonstrates variants for each patient along with the platform associated with their detection and stratification of variants by molecular characterization, fusions, WHO entity-defining criteria, prognostication, potentially actionable target, and diagnostic refinement.

Clinically pertinent findings (tier 1 and/or tier 2) were detected in 49/53 (92%) cases from one or more of the broad platforms employed, namely WES, RNA-seq, and/or methylation profiling (Figure 5E; Supplementary Table 1). 65/84 (65%) of the most clinically relevant calls listed in Supplementary Table 1 would not have been detected by our current in-house conventional assays, including IHC, fluorescence in situ hybridization (FISH), or our targeted DNA/RNA-based NGS panel (the TSO500 panel). Diagnostic refinement via methylation profiling, RNA-seq-based transcriptional readouts providing outlier expression data for potential therapeutic exploitation, and copy number calls with prognostic relevance accounted for 51/55 (93%) of these added-value calls. The remaining 4 calls included less frequent fusion variants not covered by the TSO500 panel, including TPR-NTRK1, KIF21B-NTRK1, PTPRZ1-MET, and MYBL1-KHDRBS3 fusion variants, and a PTCH1 germline variant that is not covered by the targeted NGS panel. Those calls that would have been covered primarily represented SNVs in well-described oncogenes or tumor suppressors (covered by the TSO500 panel), common fusion variants such as BRAF-KIAA1549 in pilocytic astrocytoma (covered by the TSO500 panel’s RNA component) or CNAs as assessed by FISH currently at our institution (eg, 1q analysis in ependymomas using the same FISH assay that is also commonly used in the workup of oligodendroglioma). A summary of these results and how each platform contributed to WHO entity-defining criteria or other diagnostic refinement, independently prognostic information, and/or therapeutic targeting for each diagnostic class is shown in Figure 5. For closer inspection, the platform-dependent contributions are further highlighted in Supplementary Figure 2 for ependymomas, medulloblastomas, pilocytic astrocytomas, and infiltrating astrocytomas.

### Towards Complete Assessment: Molecular Profiling, Tumor Modeling, and Autopsy

**Figure 6.**
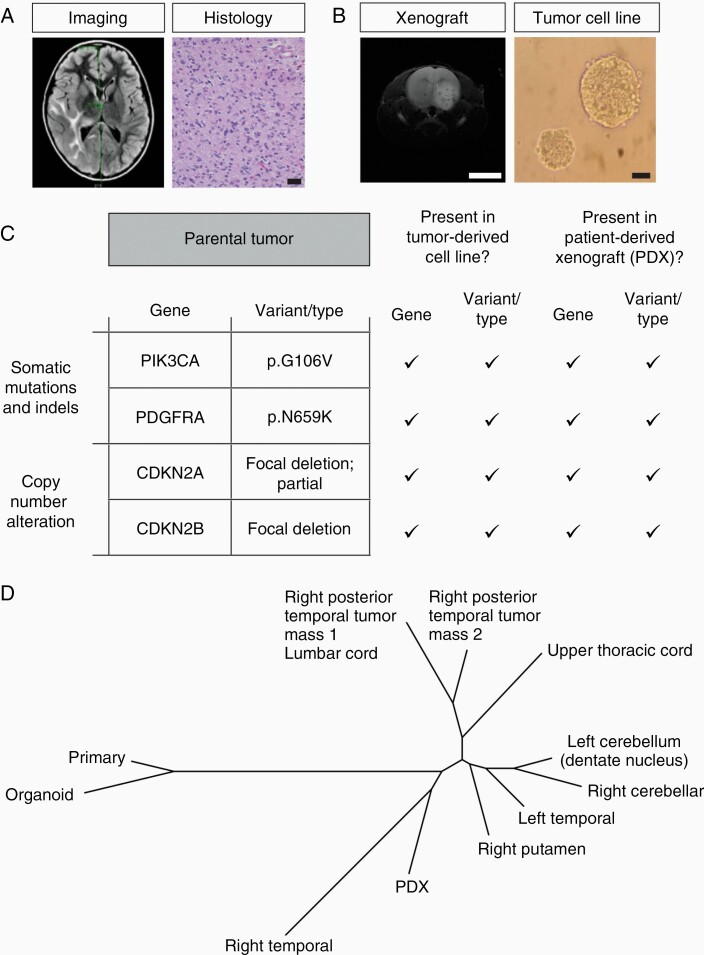
Sample case assessment. (A) T2-weighted axial MRI demonstrating multi-lobular infiltrative disease and diagnosed as gliomatosis cerebri with histology and clinical findings (scale bar = 50 µm). (B) Upon initial biopsy, a tumor-derived cell line (right, scale bar = 50 µm) was propagated along with a patient-derived xenograft (PDX, left, scale bar = 4800 µm) allowing us to model disease. (C) WES was performed on parental tumor, tumor-derived cell line, and the PDX demonstrating targetable somatic alterations that were detected with initial interrogation of the parental tumor. (D) Phylogenetic tree that demonstrates tumor evolution and variants detected from materials sequenced from initial biopsy to autopsy which showed dissemination of disease to the spine.

A future direction of the program is to integrate molecular profiling with tumor modeling via patient-specific cell culture and patient-derived xenograft (PDX) generation for drug screening as well as to follow patients with difficult-to-treat tumors to autopsy. To illustrate the feasibility and potential of this approach, 1 case is presented in further detail (Figure 6). To put this patient in context, an MRI of a 9-year-old boy with right cranial nerve deficits demonstrated diffuse, non-enhancing signal abnormality involving the right cerebral hemisphere with extension into the basal ganglia and thalamus (Figure 6A, left panel). Biopsy revealed a high-grade infiltrating astrocytoma with nonspecific features of angulated, hyperchromatic nuclei (Figure 6A, right panel). Tissue was allocated for WES, primary cell culture, and PDX development (Figure 6B). WES demonstrated somatic alterations in targetable genes including *PIK3CA* and *PDGFRA* and loss of *CDKN2A/B*. Of note, these same variants were maintained in both the tumor-derived cell line and PDX, demonstrating the genomic integrity of these model platforms (Figure 6C). Despite treatment, the patient’s tumor progressed, and tissues were altruistically donated via autopsy to permit further study of the tumor. A phylogeny tree demonstrated primary tumor evolution and variants detected from materials sequenced from initial biopsy to autopsy, which showed dissemination of disease to the spine (Figure 6D).

## Discussion

Pediatric patients with neoplasms of the CNS represent a challenging group of patients with a complex and diverse set of histologically, molecularly, and clinically heterogeneous features. Developing a program that can accommodate all patients seamlessly and provide the best available care for each individual patient requires a set of tools that are robust, flexible, and comprehensive. Here, we sought to determine whether the incorporation of high-throughput assays with broad genomic assessment adds value to the workup of pediatric tumors. All 53 patients enrolled in our study were offered WES along with matched non-neoplastic (germline) sequencing. About half of the patients were additionally offered RNA-seq (n = 28) and/or DNA methylation array profiling (n = 27) based on tissue availability. In order to assess the clinical yield of the detected alterations, we assigned each variant to 1 of the 3 tiers: tier 1 variants were considered targetable by currently available, well-established drugs in at least 1 tumor type (eg, BRAF V600E), or were considered diagnostic WHO entity-defining criteria (eg, C19MC amplification); tier 2 variants were of additional prognostic value independent of the diagnosis itself (eg, 1a gain in ependymoma), demonstrated likely functional relevance in the tumor either because they were characteristic of a particular tumor type and/or occurred in known cancer genes (eg, BRAF fusion transcripts); tier 3 variants were of undetermined significance. For 84 of the most clinically pertinent calls over 49 cases (outlined in Supplementary Table 1), we determined that 65% would not have been covered by our current standard in-house assays (including IHC, FISH, or the TSO500 panel). Those alterations providing the most yield over and above these assays included increasing diagnostic precision with methylation profiling, increasing prognostic information via copy number analysis afforded by WES, and by developing an orthogonal transcriptional readout via bulk RNA-seq that revealed outliers with the potential for guiding therapeutic options.

In contrast to copy number assessment, WES rarely provided additional benefit with respect to SNV/INDEL detection over and above targeted NGS. In particular, solid tumor-targeted panels are specifically designed to cover clinically relevant SNVs that might be of therapeutic relevance, including *BRAF* V600E, which was the most common high-yield SNV detected in this diverse cohort (5/53 cases) by WES. To illustrate the importance of V600E assessment by WES or other means, in 1 case (patient P53), a 12-year-old male with pilocytic astrocytoma was found to harbor *BRAF* V600E mutation with 13% variant allele frequency (VAF) via WES. Of note, given that *BRAF* V600E is much less common in histologically defined pilocytic astrocytomas than BRAF tandem duplication, it was of critical importance to assess for this variant despite a lower pretest probability given the histology. Postoperatively, the patient commenced treatment with Dabrafenib, and within 2 months of targeted therapy, imaging demonstrated decreased enhancing tumor burden (Supplementary Figure 3). The patient remained on Dabrafenib for 13 months with no evidence of disease progression radiographically.

WES detected an important *PTCH1* germline variant in patient P46 with an SHH-activated medulloblastoma, which is not covered by the TSO500. This finding also stresses the clinical value of germline assessment in some cases, which is not routinely performed in the context of our in-house targeted panel. WES does detect variants that while not of direct clinical relevance prompt consideration for further analysis in the research context. In some cases, robust variants of undetermined significance were detected which will be catalogued for future study. These included a truncating *SRGAP3* mutation in a case without any clinically relevant calls by any modality and with a poorly characterized histological diagnosis (neuroepithelial neoplasm, NOS). A caveat to WES that should be noted is that depending on the assay design, the depth of coverage of certain regions, some of the crucial diagnostic importance, may be considerably lower than more targeted assays. In our own cohort, for example, an *H3F3A* K27M mutation was missed by WES for patient P7 due to very low depth of coverage for that locus, and the variant was subsequently picked up both by a stand-alone PCR as well as by IHC using a commercially available antibody with high sensitivity and specificity.

Methylation profiling yielded clinically impactful diagnostic information in 18/27 cases, which in part reflects the increasing incorporation of tumor subclassification into the WHO diagnostic rubric. For all ependymomas and medulloblastomas where methylation was employed, profiling was able to subclassify tumors into either PFA or PFB subtypes of ependymoma (WHO recognized entities as of 2021) and was able to subclassify medulloblastomas into either WNT-activated, SHH-activated, or non-WNT/non-SHH-activated subtypes (as mandated by the WHO since 2016). In 1 salient case (P42; Figure 4), the methylation profile suggested with high confidence a diagnosis of pineoblastoma that was distinct from the diagnosis of medulloblastoma that was rendered by conventional means by 3 independent institutions. Of note, the histology in this case was one of a nonspecific CNS embryonal tumor, and in this setting was classified as medulloblastoma primarily because of its anatomical location (within the fourth ventricle, and with no apparent involvement of the pineal gland as shown in Supplementary Figure 4). In the absence of histopathological specificity (as might be observed in, say, the desmoplastic nodular variant of medulloblastoma) molecular information such as the methylation profile becomes more important for pathological interpretation. Indeed, within the section on medulloblastomas, the WHO classification now states “a combination of molecular analysis (e.g. DNA methylation profiling) and morphological interpretation provides optimal prognostic and predictive information.” ^40^

Even when the classifier was unable to provide an exact match, the slightly lower calibrated scores themselves, or indeed a failure to match at all gave a perhaps counterintuitive confidence to pathologists and clinicians that they were in fact dealing with an unusual tumor and that a correct conventional diagnosis was not merely being missed.

Not only did RNA-seq detect common fusions such as BRAF-KIAA1549 in 4 pilocytic astrocytomas, but it also detected rarer fusions including 2 cases with NTRK fusions, and one with a clear MYBL1 driver in an infiltrating astrocytoma. Given the recent FDA approval for Larotrectinib, an oral, brain-penetrating TRK inhibitor, detection of fusions involving NTRK genes may serve a critical role in treatment strategy moving forward. RNA-seq also yielded data on outlier gene expression with the potential to inform therapeutic regimens in 23/28 cases, representing an exciting and understudied opportunity. Because outlier expression data were not necessarily predicted by the histological subtype of the tumor or other orthogonal diagnostic methods but for this reason potentially reveals an important and more therapeutically relevant readout, we note that the use of transcriptional data for this purpose and the clinical outcomes associated with such use requires further in-depth study, longer-term clinical follow-up, and ideally randomized trials to determine efficacy.

We therefore envision a complement of assays that as a foundation includes a targeted DNA/RNA panel with deep coverage and methylation profiling. If no likely oncogenic drivers are found, further investigation via RNA-based fusion detection platforms and WES would be recommended. Finally, while the use of a transcriptional readout from bulk RNA-seq to inform therapeutic decision making requires further in-depth study for validation, the data provided here show that RNA-seq has significant add-on potential to revolutionize patient care and merits further research.

This investigation is one of the first to examine the combined clinical utility of using WES, RNA-seq, and methylation profiling within the framework of clinical management of pediatric patients with CNS tumors. Similar combined approaches have also recently been demonstrated by the ZERO Childhood Cancer Program, which was a multi-institutional, national trial including all pediatric cancers.^41^ As we continue to evaluate and refine precision medicine approaches, evaluating longitudinal data is critical for patients who had their care redirected due to the detection of clinically relevant variants, and how this redirection affected their clinical outcome. This is the focus of ongoing studies including the TARGET initiative by the Children’s Oncology Group as well as the NCI-pediatric MATCH trial,^42^ but more focused trials in the field of pediatric CNS tumors are needed to discern the clinical utility of these trials in this distinct population. We have demonstrated our ability to detect clinically relevant alterations consistently using non-conventional, broad assays. As the field of precision medicine evolves, tracking the real-world clinical impact of particular variants and the assays used for their detection will be crucial to standardize diagnostics and treatment opportunities across centers that care for our most vulnerable patients.

## Supplementary Material

vdac031_suppl_Supplementary_Material
