# Supplementary material for: Utility of multimodality molecular profiling for pediatric patients with central nervous system tumors
Source: Neurooncol Adv. 2022 Mar 10;4(1):vdac031. doi: 10.1093/noajnl/vdac031 (PMC9034114; doi:10.1093/noajnl/vdac031)
Supplement: vdac031_suppl_Supplementary_Material [file vdac031_suppl_supplementary_material.docx]

**Supplementary Table 1**. Summary of included patients and critical calls.

| STUDY ID | DIAGNOSTIC CLASS | MOST CLINICALLY RELEVANT CALLS - DESCRIPTION | TIER | PLATFORM | CALL TYPE | FUSION DRIVER | WHO ENTITY DEFINING | PRIMARILY PROGNOSTIC | POTENTIAL TREATMENT TARGET | DIAGNOSTIC AIDE OR REFINED DIAGNOSIS | ALTERATION IS COVERED BY EITHER CURRENT IN-HOUSE TARGETED NGS PANEL (WHICH INCLUDES LIMITED FUSION DETECTION), FISH, OR IHC |
| --- | --- | --- | --- | --- | --- | --- | --- | --- | --- | --- | --- |
| 3 | Ependymoma | Classifier: Ependymoma, posterior fossa group A | 1 | METH | METH CLASS |  | X |  |  |  | NO |
| 4 | Infiltrating astrocytoma | Classifier: No match. Glioblastoma, IDH wildtype, score of 0.88. | 2 | METH | METH CLASS |  |  |  |  | X | NO |
| 9 | Ependymoma | Classifier: Ependymoma, posterior fossa group A | 1 | METH | METH CLASS |  | X |  |  |  | NO |
| 11 | Ependymoma | Classifier: Ependymoma, posterior fossa group B | 1 | METH | METH CLASS |  | X |  |  |  | NO |
| 12 | Ependymoma | Classifier: Ependymoma, posterior fossa group A | 1 | METH | METH CLASS |  | X |  |  |  | NO |
| 13 | Medulloblastoma | Classifier: Medulloblastoma, subclass group 3 | 1 | METH | METH CLASS |  | X |  |  |  | NO |
| 24 | Medulloblastoma | Classifier: Medulloblastoma, subclass SHH B (infant) | 1 | METH | METH CLASS |  | X |  |  |  | NO |
| 25 | Infiltrating astrocytoma | Classifier: Glioblastoma, IDH wildtype | 1 | METH | METH CLASS |  |  |  |  | X | NO |
| 26 | CNS Embryonal Tumor | Classifier: No match. FOXR2 Embryonal Tumor with score of 0.717. | 2 | METH | METH CLASS |  |  |  |  | X | NO |
| 27 | Infiltrating astrocytoma | Classifier: No match. Closest match is GBM, IDH-WT, RTKII | 2 | METH | METH CLASS |  |  |  |  | X | NO |
| 28 | Medulloblastoma | Classifier: Medulloblastoma, subclass group 4 | 1 | METH | METH CLASS |  | X |  |  |  | NO |
| 34 | Medulloblastoma | Classifier: Medulloblastoma, subclass group 4 | 1 | METH | METH CLASS |  | X |  |  |  | NO |
| 40 | Medulloblastoma | Classifier: Medulloblastoma, WNT | 1 | METH | METH CLASS |  | X |  |  |  | NO |
| 41 | Pilocytic Astrocytoma | Classifier: Posterior fossa pilocytic astrocytoma | 1 | METH | METH CLASS |  |  |  |  | X | NO |
| 42 | Medulloblastoma | Classifier: Pineoblastoma | 1 | METH | METH CLASS |  |  |  |  | X | NO |
| 43 | Pilocytic Astrocytoma | Classifier: Posterior fossa pilocytic astrocytoma | 1 | METH | METH CLASS |  |  |  |  | X | NO |
| 46 | Medulloblastoma | Classifier: Medulloblastoma, subclass SHH B (infant) | 1 | METH | METH CLASS |  | X |  |  |  | NO |
| 53 | Pilocytic Astrocytoma | Classifier: low grade glioma, subclass midline pilocytic astrocytoma | 1 | METH | METH CLASS |  |  |  |  | X | NO |
| 7 | Infiltrating astrocytoma | H3F3A K27M | 1 | PCR, IHC | SNV |  | X |  |  |  | YES |
| 6 | Infiltrating astrocytoma | Expression: NCOA2, PTPN11, KDR, JAK2 | 2 | RNAseq | EXPRESSION |  |  |  | X |  | NO |
| 12 | Ependymoma | Expression: PIK3R1 | 2 | RNAseq | EXPRESSION |  |  |  | X |  | NO |
| 13 | Medulloblastoma | Expression: DNMT3A, ABL1, RARA | 2 | RNAseq | EXPRESSION |  |  |  | X |  | NO |
| 20 | Other: High grade neuroepithelial neoplasm, NOS | Expression: JAK2, PTCH1 | 2 | RNAseq | EXPRESSION |  |  |  | X |  | NO |
| 22 | Medulloblastoma | Expression: DNMT3A, CDKN1B | 2 | RNAseq | EXPRESSION |  |  |  | X |  | NO |
| 25 | Infiltrating astrocytoma | Expression: PDGFRA, CDK6, FGFR3 | 2 | RNAseq | EXPRESSION |  |  |  | X |  | NO |
| 26 | CNS Embryonal Tumor | Expression: CDKN1B, CCND2 | 2 | RNAseq | EXPRESSION |  |  |  | X |  | NO |
| 27 | Infiltrating astrocytoma | Expression: CDK4, CCND2 | 2 | RNAseq | EXPRESSION |  |  |  | X |  | NO |
| 28 | Medulloblastoma | Expression: GPHN | 2 | RNAseq | EXPRESSION |  |  |  | X |  | NO |
| 29 | Infiltrating astrocytoma | Expression: PDGFRA | 2 | RNAseq | EXPRESSION |  |  |  | X |  | NO |
| 30 | Pilocytic Astrocytoma | Expression: PDFGRA | 2 | RNAseq | EXPRESSION |  |  |  | X |  | NO |
| 32 | Other: Pleormorphic xanthoastrocytoma | Expression: ATP1A1 | 2 | RNAseq | EXPRESSION |  |  |  | X |  | NO |
| 33 | Pilocytic Astrocytoma | Expression: PIK3R1 | 2 | RNAseq | EXPRESSION |  |  |  | X |  | NO |
| 34 | Medulloblastoma | EXPRESSION: RAF1, ACVR1 | 2 | RNAseq | EXPRESSION |  |  |  | X |  | NO |
| 35 | Germ Cell Tumor | Expression: SMO, PDGFRA, RARA, FGFR1, ABL1, PDGFRB | 2 | RNAseq | EXPRESSION |  |  |  | X |  | NO |
| 36 | Pilocytic Astrocytoma | Expression: PDGFRA | 2 | RNAseq | EXPRESSION |  |  |  | X |  | NO |
| 38 | Craniopharyngioma | Expression: NFKB2, FGFR1, JUN | 2 | RNAseq | EXPRESSION |  |  |  | X |  | NO |
| 39 | Pilocytic Astrocytoma | Expression: FCGR2B | 2 | RNAseq | EXPRESSION |  |  |  | X |  | NO |
| 41 | Pilocytic Astrocytoma | Expression: JUN, RARA, BCR | 2 | RNAseq | EXPRESSION |  |  |  | X |  | NO |
| 45 | Craniopharyngioma | Expression: *CDKN2B* | 2 | RNAseq | EXPRESSION |  |  |  | X |  | NO |
| 46 | Medulloblastoma | Expression: NTRK1, PTCH1 | 2 | RNAseq | EXPRESSION |  |  |  | X |  | NO |
| 48 | Pilocytic Astrocytoma | Expression: RARA | 2 | RNAseq | EXPRESSION |  |  |  | X |  | NO |
| 52 | Other: Spinal glioma, NOS | Expression: RARA, NTRK1, JUN | 2 | RNAseq | EXPRESSION |  |  |  | X |  | NO |
| 25 | Infiltrating astrocytoma | Fusion: TPR-NTRK1, and PTPRZ1--MET | 1 | RNAseq | FUSION | X |  |  | X | X | NO |
| 29 | Infiltrating astrocytoma | Fusion: MYBL1-KHDRBS3 | 1 | RNAseq | FUSION | X | X |  |  |  | NO |
| 30 | Pilocytic Astrocytoma | Fusion: KIAA1549-BRAF | 2 | RNAseq | FUSION | X |  |  |  | X | YES |
| 33 | Pilocytic Astrocytoma | Fusion: KIAA1549-BRAF | 2 | RNAseq | FUSION | X |  |  |  | X | YES |
| 43 | Pilocytic Astrocytoma | Fusion: KIAA1549-BRAF | 2 | RNAseq | FUSION | X |  |  |  | X | YES |
| 48 | Pilocytic Astrocytoma | Fusion: KIAA1549-BRAF | 2 | RNAseq | FUSION | X |  |  |  | X | YES |
| 52 | Other: Spinal glioma, NOS | Fusion: KIF21B-NTRK1 | 1 | RNAseq | FUSION | X |  |  | X | X | NO |
| 2 | Ependymoma | Positive for 1q gain | 2 | WES | CNA |  |  | X |  |  | YES |
| 3 | Ependymoma | Positive for 1q gain | 2 | WES | CNA |  |  | X |  |  | YES |
| 5 | Other: Poorly differentiated chordoma | SMARCB1 loss | 2 | WES | CNA |  |  |  |  | X | NO |
| 9 | Ependymoma | Positive for 1q gain | 2 | WES | CNA |  |  | X |  |  | YES |
| 10 | Ependymoma | Negative for 1q gain | 2 | WES | CNA |  |  | X |  |  | N/A |
| 11 | Ependymoma | Negative for 1q gain | 2 | WES | CNA |  |  | X |  |  | NO |
| 12 | Ependymoma | Positive for 1q gain | 2 | WES | CNA |  |  | X |  |  | YES |
| 13 | Medulloblastoma | Negative for iso(17q) and negative for MYC amplification | 2 | WES | CNA |  |  | X |  |  | NO |
| 14 | Ependymoma | Positive for 1q gain | 2 | WES | CNA |  |  | X |  |  | YES |
| 18 | Infiltrating astrocytoma | *EGFR* amplification | 2 | WES | CNA |  |  |  | X | X | YES |
| 22 | Medulloblastoma | Positive for iso(17q) | 2 | WES | CNA |  |  | X |  |  | NO |
| 23 | Medulloblastoma | Gain of chromosome 17 | 2 | WES | CNA |  |  | X |  |  | NO |
| 24 | Medulloblastoma | Negative for GLI2 amplification and negative for 14q loss | 2 | WES | CNA |  |  | X |  |  | NO |
| 27 | Infiltrating astrocytoma | CDK4 amplification | 2 | WES | CNA |  |  |  |  | X | YES |
| 28 | Medulloblastoma | Positive for iso(17q) | 2 | WES | CNA |  |  | X |  |  | NO |
| 31 | CNS Embryonal Tumor | C19MC amplification | 1 | WES | CNA |  | X |  |  |  | NO |
| 32 | Other: Pleormorphic xanthoastrocytoma | CDKN2A/B loss | 2 | WES | CNA |  |  | X |  | X | YES |
| 34 | Medulloblastoma | Negative for loss of chromosome 11 and negative for gain of chromsome 17 | 2 | WES | CNA |  |  | X |  |  | NO |
| 40 | Medulloblastoma | Monosomy of chromosome 6 | 2 | WES | CNA |  |  |  |  | X | NO |
| 4 | Infiltrating astrocytoma | EGFR p.P772_nofs +CCA | 2 | WES | INDEL |  |  |  | X | X | YES |
| 15 | Infiltrating astrocytoma | *NF1* D2284_fs | 2 | WES | INDEL |  |  |  |  | X | YES |
| 46 | Medulloblastoma | *PTCH1* T18_fs | 1 | WES | INDEL |  |  |  |  | X | NO |
| 19 | Pilocytic Astrocytoma | No clinically relevant alterations detected | 0 | WES | N/A |  |  |  |  |  | N/A |
| 1 | Infiltrating astrocytoma | *PIK3CA* H1047R | 2 | WES | SNV |  |  |  | X | X | YES |
| 16 | Infiltrating astrocytoma | *TP53* c.375+1G>A | 2 | WES | SNV |  |  |  |  | X | YES |
| 21 | Infiltrating astrocytoma | EGFR G598A and EGFR P596L | 2 | WES | SNV |  |  |  | X | X | YES |
| 32 | Other: Pleormorphic xanthoastrocytoma | *BRAF* V600E | 1 | WES | SNV |  |  |  | X | X | YES |
| 35 | Germ Cell Tumor | *KIT* D816V | 2 | WES | SNV |  |  |  |  | X | YES |
| 37 | Craniopharyngioma | *CTNNB1* S33Y | 2 | WES | SNV |  |  |  |  | X | YES |
| 38 | Craniopharyngioma | *CTNNB1* S33Y | 2 | WES | SNV |  |  |  |  | X | YES |
| 40 | Medulloblastoma | *CTNNB1* S37Y | 1 | WES | SNV |  |  |  |  | X | YES |
| 44 | Other: Choroid plexus carcinoma | *NF1* D2465E, *PER1* P805C | 2 | WES | SNV |  |  |  |  | X | YES |
| 45 | Craniopharyngioma | *CTNNB1* S37F | 2 | WES | SNV |  |  |  |  | X | YES |
| 47 | Infiltrating astrocytoma | *BRAF* V600E | 1 | WES | SNV |  |  |  | X | X | YES |
| 49 | Other: Low grade astrocytoma, NOS | *BRAF* V600E | 1 | WES | SNV |  |  |  | X | X | YES |
| 51 | Other: Astrocytoma, NOS | *BRAF* V600E | 1 | WES | SNV |  |  |  | X | X | YES |
| 53 | Pilocytic Astrocytoma | *BRAF* V600E | 1 | WES | SNV |  |  |  | X | X | YES |
| 17 | Other: Neuroepithelial neoplasm, NOS | SRGAP3 S939X | 3 | WES, METH | N/A |  |  |  |  |  | N/A |
| 50 | Germ Cell Tumor | No clinically relevant alterations detected | 0 | WES, METH | N/A |  |  |  |  |  | N\A |
| 8 | CNS Embryonal Tumor, NOS | No clinically relevant alterations detected | 0 | WES, RNASeq, METH | N/A |  |  |  |  |  | N\A |

**Supplementary Figure 1.** *Principal Component Analysis.* Methylation profiling data generated from 25 patients was used to perform principal component analyses (PCA). Based on methylation patterns alone, this cohort of patients with CNS tumors demonstrated histological clustering of disparate entities.


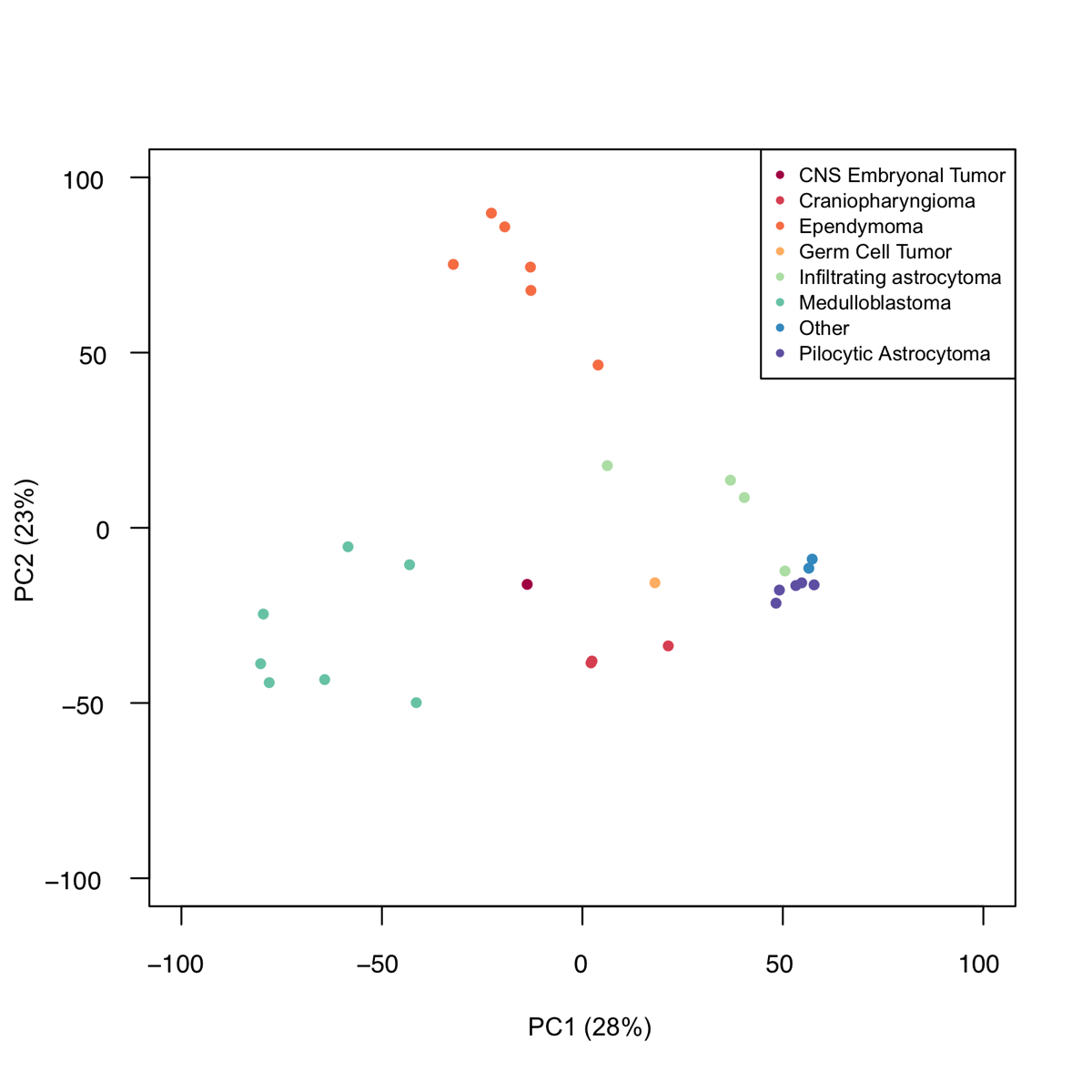


**
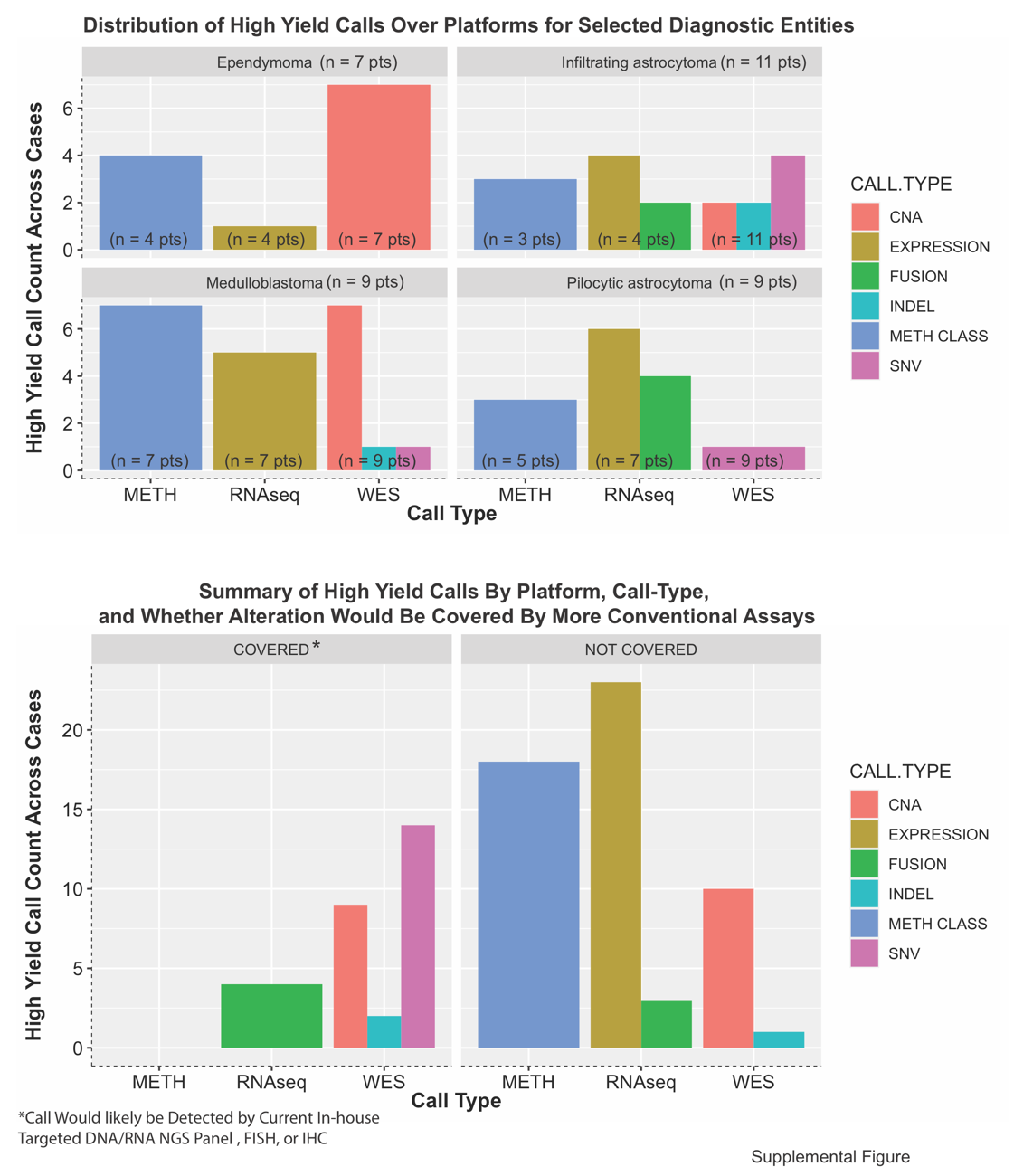
Supplementary Figure 2**. Summary of how each sequencing platform contributed to high yield diagnostic calls for selected diagnostic entities.

**
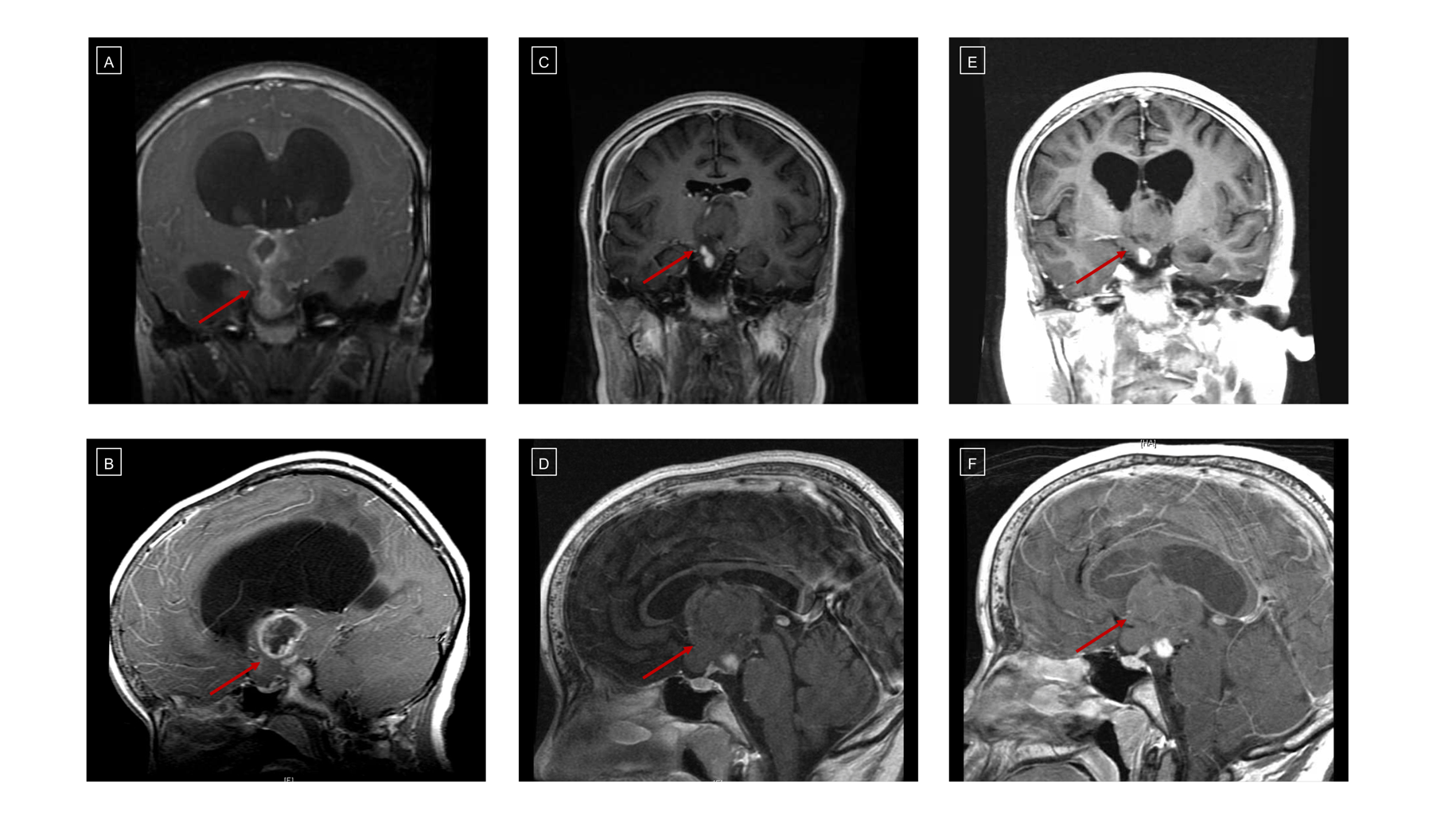
Supplementary Figure 3**. *Illustrative case*. Representative coronal and sagittal T1 + gadolinium contrast magnetic resonance (MR) images from a selected patient that received Dabrafenib, a BRAF inhibitor, based on integrated molecular analysis. A and B. MRI at diagnosis demonstrated contrast enhancing suprasellar mass causing hydrocephalus. Patient was treated with endoscopic biopsy and ventriculoperitoneal shunt placement. Pathology was consistent with a pilocytic astrocytoma and integrated molecular analysis demonstrated a BRAF V600E mutation. The patient was started on Dabrafenib five months after surgery. C and D. MR imaging two months after beginning Dabrafenib treatment demonstrated decreased enhancement within the suprasellar tumor. E and F. Repeat imaging 13 months after beginning BRAF targeted treatment demonstrating stable tumor size.

**Supplementary Figure 4.** Representative image of preoperative MRI for patient P42. In this sagittal 3D T1 CUBE C+ sequence, a mass is seen centered within the fourth ventricle (long arrow) with impingement upon the brainstem, and without a radiologically evident connection to the region of the pineal gland (short arrow). The tumor was diagnosed based on histopathology as a medulloblastoma. The methylation profile was a match for pineoblastoma group B with a calibrated score of 0.99.
